# The Need for More Prehospital Research on Language Barriers: A Narrative Review

**DOI:** 10.5811/westjem.2015.8.27621

**Published:** 2015-12-08

**Authors:** Ramsey C. Tate

**Affiliations:** University of New Mexico, Department of Emergency Medicine, Albuquerque, New Mexico

## Abstract

**Introduction:**

Despite evidence from other healthcare settings that language barriers negatively impact patient outcomes, the literature on language barriers in emergency medical services (EMS) has not been previously summarized. The objective of this study is to systematically review existing studies of the impact of language barriers on prehospital emergency care and identify opportunities for future research.

**Methods:**

A systematic review with narrative synthesis of publications with populations specific to the prehospital setting and outcome measures specific to language barriers was conducted. A four-prong search strategy of academic databases (PubMed, Academic Search Complete, and Clinical Key) through March 2015, web-based search for gray literature, search of citation lists, and review of key conference proceedings using pre-defined eligibility criteria was used. Language-related outcomes were categorized and reported as community-specific outcomes, EMS provider-specific outcomes, patient-specific outcomes, or health system-specific outcomes.

**Results:**

Twenty-two studies met eligibility criteria for review. Ten publications (45%) focused on community-specific outcomes. Language barriers are perceived as a barrier by minority language speaking communities to activating EMS. Eleven publications (50%) reported outcomes specific to EMS providers, with six of these studies focused on EMS dispatch. EMS dispatchers describe less accurate and delayed dispatch of resources when confronted with language discordant callers, as well as limitations in the ability to provide medical direction to callers. There is a paucity of research on EMS treatment and transport decisions, and no studies provided patient-specific or health system-specific outcomes. Key research gaps include identifying the mechanisms by which language barriers impact care, the effect of language barriers on EMS utilization and clinically significant outcomes, and the cost implications of addressing language barriers.

**Conclusion:**

The existing research on prehospital language barriers is largely exploratory, and substantial gaps in understanding the interaction between language barriers and prehospital care have yet to be addressed. Future research should be focused on clarifying the clinical and cost implications of prehospital language barriers.

## INTRODUCTION

Emergency medical services (EMS) systems operate in multicultural environments. Language discordance between providers and patients in the prehospital setting occurs frequently. More than 20% of households in the United States report a home language other than English, and limited-English proficiency (LEP) speakers are a rapidly growing population.^1^ EMS providers deliver care in chaotic and dynamic situations, such as at the scene of a collision on a roadside or in a patient’s home surrounded by distressed family members. EMS providers rely on accurate and efficient communication to ensure personal safety, rapidly assess patients, and make decisions about appropriate care. Language barriers heighten the uncertainty of EMS work.

The deleterious impact of language barriers on medical care has been widely documented in outpatient and hospital-based settings, including increased rates of communication errors, unnecessary invasive procedures and testing, and increased costs of care.^2^–^7^ However, the impact of language barriers is less well-understood in the prehospital setting and the literature has not been previously reviewed.^8^–^12^ The objective of this systematic review with narrative synthesis is to summarize the existing literature on the impact of language barriers on prehospital care and identify opportunities for future research.

## METHODS

### Search Strategy

Publications were identified through a four-prong, sequential search strategy: 1) database searches, 2) web-based search, 3) citation searches, and 4) review of conference proceedings. Both published and gray literature were searched to identify all relevant research. Gray literature includes a variety of document types, such as theses or posters, collected and maintained by libraries or institutional repositories but which are not commercially published.^13^ The search strategy was reviewed by a research librarian at the University of New Mexico Health Sciences Library & Informatics Center to refine search terms.

1. Database searches*:* Three primary academic databases, PubMed/Medline, Clinical Key, and Academic Search Complete, were searched to identify relevant publications. PubMed/Medline (1966–March 2015) was searched using the MeSH terms “emergency medical services” and “communication barriers” with no further limits applied. ClinicalKey (2004–March 2015) was searched using the terms “prehospital and language barriers or EMS and language barriers” with source type restricted to Medline abstracts, full text articles, and clinical trials. Academic Search Complete (1965–March 2015) was searched using the subject terms “emergency medical services” and “language” with no further limits applied.
2. Web-based search*:* Google Scholar was searched using the terms “prehospital language barrier” and then searched again using the terms “EMS language barrier” with the additional restriction of excluding patents. The first 150 results as ranked by relevance were evaluated for each search term.
3. Citation searches*:* Each individual citation within the reference lists of reviewed publications was searched in Thomson Reuters’ Web of Science for related citations.
4. Review of conference proceedings: Three annual conferences were identified as the most likely locations for presentations of research on prehospital language barriers that may not have yet been published. PubMed/Medline includes the indexed abstracts for the American College of Emergency Physicians annual conference. Abstracts from the annual conference proceedings for the Society of Academic Emergency Medicine and National Association of EMS Professionals were reviewed from 2010 to 2014 to identify research that may be too recent to have been published in peer-reviewed journals.

### Study Selection

**Figure 1:**
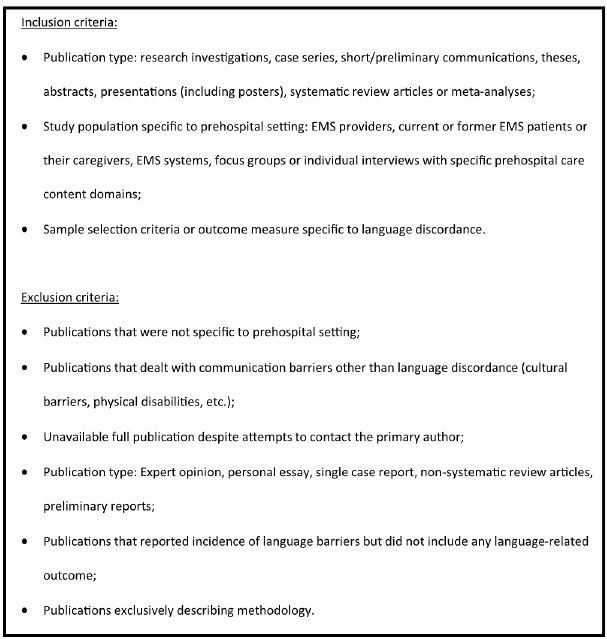
Inclusion and exclusion criteria of publications reviewed with regard to language barriers and use of emergency medical services in the United States. *EMS,* emergency medical services

Titles and abstracts of publications were reviewed to determine whether publications met initial inclusion and exclusion criteria. After an initial screening of the abstract, publications that were potentially eligible were then reviewed in their entirety for inclusion and exclusion criteria (Figure 1). For publications that did not have abstracts available, the complete publications were reviewed to ascertain eligibility. If full publications were not available even after attempting to contact the primary author, they were excluded from the review. There were no exclusion criteria by language of publication or by date of publication.

### Data Extraction and Quality Assessment

The data extraction tool included the following fields: unique study identifier, author, date of publication, research design, study sample characteristics, EMS stakeholder groups studied (minority language speaking communities, EMS providers, or health system), country of study, key results, key limitations, and eligibility for inclusion in review. All publications that met potential eligibility criteria after abstract review were included in the data extraction tool on review of the full publication. We reviewed eligible studies using the Critical Appraisal Skills Framework Programme appraisal tools for qualitative studies, cohort studies, and case-control studies.^14^ Survey studies were reviewed using the Center for Evidence-Based Management critical appraisal tool.^15^ The Mixed Methods Appraisal Tool was used to review mixed methods studies.^16^ Results and methods are reported in accordance with the Preferred Reporting Items for Systematic Reviews and Meta-Analyses recommendations.^17^,^18^

### Outcomes of Interest and Data Synthesis

A narrative synthesis of the results was planned prior to implementation of the literature search due to anticipated heterogeneity of outcome measures.^19^ Language-related outcomes from each publication were categorized as 1) community-specific outcomes, defined as measures of LEP community members’ knowledge about EMS, trust in EMS, or confidence in their ability to activate EMS; 2) EMS provider-specific outcomes, defined as measures of stress, provider self-efficacy, training, or decision-making; 3) patient-specific outcomes, defined as measures of patient satisfaction or specific clinical outcome measures; or, 4) health system-specific outcomes, defined as measures of cost, quality, or efficiency.

## RESULTS

**Figure 2:**
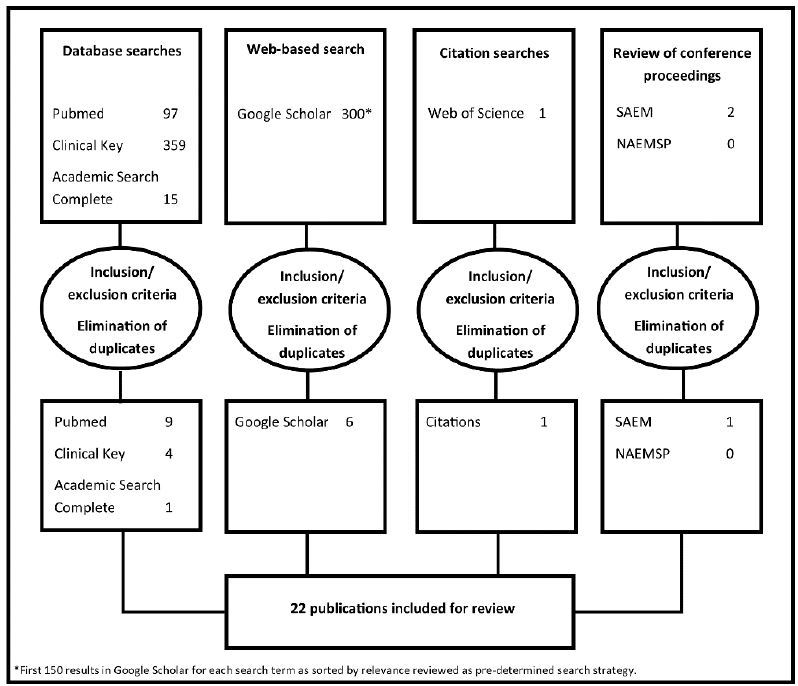
The four-pronged search strategy identified 22 publications for review.

A total of 22 publications were identified as meeting inclusion and exclusion criteria and are reviewed in the results.^8^–^12^,^20^–^36^ (Figure 2 and Table) A single prior systematic review of the literature was identified.^20^ However, this review of the barriers and facilitators of EMS utilization by minority ethnic communities was broader than the specific question of the impact of language barriers on prehospital care. This review was unpublished outside of a poster presentation, unable to be replicated from the methodology, and a full list of citations was unavailable.

The remaining 21 publications offer insight into the mechanisms by which language barriers impact EMS care and provide an outline for future research in prehospital language barriers.

### Language Barriers Impede Minority-Language Speaking Community Engagement with EMS

Language discordance is a perceived barrier to using EMS in the United States, the only country in which studies of engagement of minority-language speaking communities with EMS have been conducted. Focus group interviews of LEP Chinese speakers in King County, Washington, found that Chinese adults are more likely to rely upon themselves and their community in an emergency rather than on EMS. Participants in these focus groups identified language barriers as a negative factor impacting their likelihood of using EMS for emergencies while awareness of interpreter services was a potential facilitator.^28^,^36^ When members of this Chinese community were presented with hypothetical emergency scenarios, non-English speaking Chinese adults reported lower likelihood of activating EMS than Chinese adults who could speak some English.^35^ Spanish-speaking parents participating in focus groups in Kansas City, Missouri, reported awareness of 9-1-1, but uncertainty around when it is appropriate to call 9-1-1. Amongst the 49 parents who participated in these focus groups, language discordance was cited as a key barrier to calling 9-1-1.^11^ Similarly, Sasson and colleagues found that Latinos in Denver, Colorado, neighborhoods with high rates of out-of-hospital cardiac arrest but poor rates of bystander CPR also identified language discordance as a barrier to calling 9-1-1 in focus group interviews.^29^ Subramaniam et al. surveyed LEP, English proficient but non-native speaking and native English speaking caregivers in a pediatric emergency department (ED) in Detroit. They reported that LEP caregivers were less aware of EMS and reported fewer activations of EMS than both non-native English-proficient and native English speakers. Nearly a third of the LEP caregivers in this study cited inability to communicate with 9-1-1 as a barrier to using EMS.^33^

These studies reflect intentions and attitudes but are not linked to EMS utilization data. Only two studies reported EMS utilization by language group. Smith and co-investigators found that, in an adult Mexican-American population in Nueces County, Texas, who presented to an ED with stroke, language was not associated with arrival by EMS.^31^ Conversely, a single Canadian hospital’s data for patients discharged with a diagnosis of acute myocardial infarction and a recorded ethnicity of Caucasian, Chinese, South Asian, Southeast Asian, or First Nations was analyzed. The investigators found that Caucasian patients were statistically significantly more likely to present to the ED by ambulance than other ethnic groups with lower English fluency.^24^ These studies are too limited to allow generalizations about the impact of language barriers on utilization of EMS by minority language speaking communities.

A few studies suggest that increased acculturation, or adopting the values and practices of the new culture in which immigrant minority language speakers settle, may moderate negative impacts of language barriers on EMS engagement. Smith and co-authors note that most Mexican-Americans in Nueces County, Texas, are second or third generation immigrants and this Hispanic population may be more acculturated than other minority language populations.^31^ Another study of Hispanics in four states also found no difference in intent to call 9-1-1 for suspected heart attack or stroke for English-speaking Hispanics compared to Spanish-speaking Hispanics, suggesting that language may not be a significant factor in EMS engagement in acculturated Hispanic communities.^22^ In specifically assessing the effects of acculturation, Meischke and colleagues found in a 2012 survey of Cambodians in King County, Washington, that increased measures of acculturation were associated with increased likelihood of calling 9-1-1 in an emergency.^26^

#### Future Research Opportunities

Although minority-language speaking communities in the U.S. are consistent in describing language discordance as a disincentive to EMS activation, further research on actual EMS utilization by minority language speaking communities is needed to bridge the gap between perception and outcomes. Additionally, the existing body of literature suggests an opportunity to improve engagement with EMS at the community level through developing evidence-based, linguistically-appropriate outreach educating minority-language speaking communities on how and when to activate EMS for an emergency.

### Language Barriers Impede Accurate and Efficient EMS Dispatch and Current Language-Assistance Resources May Not Be Well-Adapted for EMS Use

Much of the research on the impact of language barriers on EMS care has focused on dispatch, with an association described between language barriers and delayed and inaccurate dispatch. Meischke and colleagues surveyed EMS telecommunicators in King County, Washington, and found that dispatchers reported increased stress with LEP callers, as well as perceived negative impacts on the overall care delivered by the EMS system for these callers. This study also suggested that language barriers impact dispatch by demonstrating that resources were dispatched differently (Advanced Life Support vs Basic Life Support) for calls with language barriers despite similar acuity of the complaint.^8^ Meischke and colleagues further investigated the impact of language barriers on EMS dispatch in a 2013 study that demonstrated that calls with language barriers were more likely to require changes in the on-scene resources that were initially dispatched, particularly downgrades from Advanced Life Support to Basic Life Support, suggesting that dispatchers are less accurate in dispatching resources when confronted with language barriers.^9^

The impact of delayed and less accurate dispatch on patient outcomes is unclear. The only study that reported patient-specific outcomes related to language barriers at the level of dispatch was a secondary analysis of the data from a randomized controlled trial of dispatcher-assisted CPR for cardiac arrest in King County, Washington. Dispatchers took longer to recognize cardiac arrest and initiation of bystander CPR was less common if a language barrier was present. Survival to hospital discharge was also poorer among patients in which the call to EMS involved a language barrier but did not rise to the level of statistical significance.^12^ Although not tied to outcomes, a retrospective analysis of 100 cardiac arrest calls to a London EMS dispatch center also identified language barriers as one reason that dispatcher-assisted CPR was not initiated prior to the arrival of on-scene providers.^23^

Third-party telephonic interpreter services are the language assistance technology used by most EMS dispatch centers and the primary strategy that dispatchers in the King County, Washington, studies reported using to overcome language barriers. However, these studies suggest that third-party telephonic interpretation may not be an efficient tool to aid dispatch. On review of a subset of recorded calls for life-threatening conditions that featured language barriers, Meischke and colleagues found that actual use of a telephonic interpreter was less common than self-reported by dispatchers in the survey.^8^ It is possible that dispatchers are less likely to use telephonic interpreters for life-threatening complaints and only high acuity calls were reviewed by researchers. Another possible explanation is that dispatchers do not perceive that their ability to effectively dispatch is impacted by language discordance and prefer to avoid the delay associated with interpreter services. A Swedish study reviewed calls with both on-scene and dispatch provider agreement and disagreement in the priority of calls. Dispatchers in this study had a large proportion of calls with language barriers, but dispatchers specifically indicated that language barriers were not a barrier to effective dispatch and interpreter services were not used.^25^ Another review of calls with language barriers, as compared to matched language-concordant calls, found longer dispatch times with much of the difference in dispatch times attributable to connecting to the telephonic interpreter service. However, the subset of calls with language barriers in which telephonic interpretation was used was not analyzed to measure whether use of telephonic interpreters was associated with more accurate dispatch.^9^ Further investigating the role of telephonic interpretation, Meischke and co-investigators enrolled LEP adults in a randomized controlled trial of different communication strategies for providing dispatcher-assisted CPR instructions. Participants reported better understanding of CPR instructions with telephonic interpreter use, but interpreter use delayed onset of CPR by nearly two minutes and there was no improvement in quality of CPR with interpreter use.^27^

#### Future Research Opportunities

The clinical significance of statistically significant differences in the time to dispatch and the accuracy of dispatched resources has not yet been demonstrated, signaling a key gap in the existing research. Prior research has been conducted in two-tier response systems, meaning dispatchers have the capacity to choose basic or advanced resources to be dispatched to a call, and it is unclear that these findings can be extrapolated to single-tier EMS systems in which a single level of resource is available for dispatch. In two-tier EMS systems, erring on the side of dispatching advanced resources may provide a safer response to calls with language barriers. However, the impact of language barriers on the dispatch strategies of single-tier systems is unknown. Additionally, both over-triage and under-triage of prehospital resources have cost and quality of care implications in two-tier systems that are undefined, as is the cost-effectiveness of third-party telephonic interpreters. Third-party telephonic interpretation is a time-consuming and costly strategy for overcoming language barriers and the current body of evidence demonstrates an unclear benefit to the use of telephonic interpreters. Given that third-party telephonic interpretation is the most commonly provided language assistance technology for dispatchers, further research in outcomes for calls using interpreter services, the cost-effectiveness of telephonic interpreter services, and alternative language assistance strategies is warranted.

### Language Barriers Have Unclear Impacts on EMS Field Care

The impact of language barriers on treatment and transport decisions in the field has not been directly studied. However, a pair of studies suggests that EMS provider decision-making may be different for language-discordant patients. Grow et al. reviewed prehospital encounters featuring a delay in the Minnesota State Ambulance Reporting System and found that language barriers were identified as the second most common cause of delay.^10^ Intriguingly, however, the on-scene times for calls with a reported delay due to language barrier were actually *shorter* than the on-scene times for calls with no delay. A significant limitation of the study was that the “no delay” comparison group did not come from the general pool of all EMS encounters and it is unclear if these calls had atypical features that prompted EMS providers to specifically notate “no delay” in the report. Nonetheless, the shorter on-scene time hints that, in the presence of a language barrier, EMS providers may perceive more threats to timely treatment and transport at the scene and opt to rapidly transport patients to a receiving healthcare facility, a practice known colloquially as “scoop and run.” Shorter on-scene times in the presence of a language barrier were also described by Sterling and colleagues in a retrospective review of EMS encounters in New Jersey with a complaint of chest pain.^32^ Just under 2% of encounters featured a language barrier and these encounters were statistically significantly shorter than chest pain encounters without a language barrier. In contrast to these two studies finding shorter on-scene times with language barriers, a retrospective double-cohort study of EMS encounters with LEP and English-speaking patients in Albuquerque, New Mexico, did not find a statistically significant difference in on-scene times, transport times, pain scores, number of EMS interventions, or number of medications administered.^34^ The small sample size may have limited the ability to detect differences as, in this study, there was a trend towards longer on-scene and transport times for LEP patients. A significant limitation of this study was that the LEP and English-speaking patients had marked demographic differences, with LEP patients being older and more female.

No studies that directly address patient-specific or health system-specific outcomes were identified for review. However, in the development of a theoretical framework for pediatric prehospital safety events based on focus groups of EMS providers in Multnomah County, Oregon, Cottrell and co-investigators identified language barriers as a factor that contributes to pediatric prehospital safety events.^21^ Focus groups of paramedics in the United Kingdom also identified language discordance as a barrier to adherence to asthma treatment guidelines.^30^ Collectively, these studies provide indirect suggestions that EMS care differs when confronted with language barriers, but do not allow for more nuanced analysis or conclusions.

#### Future Research Opportunities

There is a paucity of research on the impact of language barriers on on-scene treatment and transport decisions, the majority of the interaction between a patient and EMS. The dearth of studies on treatments received by language-discordant patients relative to language-concordant patients and their subsequent patient-related outcomes is a glaring opportunity for future inquiry. Indeed, the targets by which to evaluate quality of prehospital care in the context of language barriers do not appear to be well-defined. Is a shorter on-scene time for language discordant patients, as demonstrated in two studies,^10^,^32^ advantageous or disadvantageous to patients? Prehospital medicine is riven by controversy regarding whether patient care is improved by shorter on-scene times as compared to more prolonged on-scene initiation of care. Furthermore, do on-scene or transport times have differential impacts depending on the acuity or type of medical complaint? In the context of this broader uncertainty about optimal strategies for patient care, it is unclear whether language discordant patients experience better or worse quality of care. Language barriers have been associated with harmful outcomes to patients and inefficient uses of healthcare resources in a variety of other medical care settings.^2^–^7^ Despite the unique challenges of providing prehospital care, it is unlikely that EMS care is unaffected by language discordance given such broadly documented disparities. However, the existing research on the impact of language barriers on prehospital care is unable to answer questions of clinical significance or healthy equity.

## DISCUSSION

In this narrative review of the impact of language barriers on prehospital care, three domains of existing research were identified related to community-specific and EMS-provider specific outcomes. Firstly, studies of minority-language speaking communities indicate that language discordance is a perceived barrier to activating EMS. Secondly, studies of EMS dispatchers describe less accurate and delayed dispatch of resources when confronted with language discordant callers, as well as limitations in the ability to provide medical direction to callers. Thirdly, studies of on-scene EMS care hint that treatment and transport decisions may differ when EMS providers are confronted with language barriers. No studies were identified that addressed patient-specific or health system-specific outcomes. The existing literature raises provocative questions about the potential impact of language barriers on the quality of prehospital care that have yet to be studied and which facilitate the development of a future research agenda. In 2006, Jacobs and colleagues presented a proposed research agenda for language barriers in healthcare, highlighting the need for research that delineates the mechanisms by which language barriers affect healthcare, evaluates the efficacy of language assistance strategies, and defines the costs of language barriers in healthcare.^37^ All three of these questions remain unanswered for prehospital medicine and, in the context of the existing literature, outline an agenda for prehospital research.

## LIMITATIONS

A key limitation to review of the literature on prehospital language barriers is the lack of consistent terminology to identify prehospital literature. The definition of “prehospital” varies in some databases and in some countries to mean emergency medical care delivered prior to hospital care or any care delivered outside of a hospital, including outpatient care. Similarly, “emergency medical services” may refer narrowly to institutions and agencies that are organized specifically for the delivery of emergency care prior to hospital care or may index more broadly to emergency medical care delivered in or out of hospital. The lack of consistent terminology may have led to the exclusion of relevant literature. Additionally, the limitations of keyword-based searching on this topic may have biased towards U.S.-based publications that use similar terminology. Alternatively, U.S.-based publications may be over-represented due to more active research in this area.

A second limitation to interpreting the existing literature is the lack of consistent reporting of the methodology for identifying minority language speakers as well as the heterogeneity of sampling approaches. The sampling strategies of the reviewed studies included self-identification, provider identification, optional documentation fields, and proxy identifiers, such as being unable to sign an English-only form. Additionally, the measures by which to identify language proficiency are not agreed upon and may vary at different points along the series of interactions between EMS and a language-discordant patient. For example, should an interaction with a caller who can communicate the patient’s location and chief complaint to a dispatcher but who lacks the language fluency to answer questions for on-scene providers be considered to have a language barrier? This hypothetical encounter would be categorized differently using the various approaches in the existing literature. The validity and generalizability of sampling strategies to identify language barriers is unclear in the EMS context in which care is delivered along a series of interactions.

An unexpected finding of this review is the predominance of a single research group, the Northwest Preparedness and Emergency Response Research Center (NWPERRC) at the University of Washington. Eight (36%) of the publications reviewed were generated from research in King County, Washington.^8^,^9^,^12^,^26^–^28^,^35^,^36^ All EMS systems practice in multi-cultural and multi-lingual communities, but the generalizability of single-site research in EMS is unclear. Themes that emerged from studies of Chinese and Cambodian communities in King County have good concordance with studies from other minority-language speaking communities in Denver, Detroit, and Kansas City. However, Meischke and colleague’s studies of dispatchers were performed in an EMS system that has a two-tier response. Many EMS systems, in contrast, are single-tier response and the findings of differential delays based on the type of dispatched resources are difficult to interpret in the context of a single-tier response system. Likewise, the training and resources available to dispatchers in King County may not be comparable to those available to dispatchers in other EMS systems. Multi-site EMS research and research in different types of EMS systems is needed to better understand the impact of language barriers on prehospital care.

## CONCLUSION

As minority-language speaking communities grow, EMS will be increasingly confronted with language barriers. This review, the first of the literature on the impact of language barriers on prehospital care, demonstrates the heterogeneity of existing research and the substantial gaps in understanding the interaction between language barriers and prehospital care that have yet to be addressed. Future research elucidating the mechanisms by which language barriers impact the care received by minority language speakers, the effect of language barriers on patient-level or health system-level outcomes, and the cost implications of addressing language barriers is needed.

## Figures and Tables

**Table:** Reviewed literature concerning language barriers and use of emergency medical services.

First author (Year)	Design	Location	Sample	Results
Review publications				
Phung (2013)	Systematic review		Studies published in English between 2003 and 2013 with barriers/facilitators for minority populations accessing prehospital emergency medical services	16 studies included, 2 from Europe and 14 from US (only 5 of the 16 studies provided in references) Single uncited study specific to language barriers
Publications describing minority language speaking community engagement with EMS				
DuBard (2006)	Survey	US (Florida, Nebraska, North Carolina, Oklahoma)	Heart Attack and Stroke Module of the 2003 Behavioral Risk Factor Surveillance System population survey administered in English or Spanish	Spanish-speaking Hispanics have less recognition of stroke or heart attack symptoms than English-speaking Hispanics (p<0.001) No difference between Spanish-speaking Hispanics and English-speaking Hispanics in intent to call 911 for suspected heart attack or stroke (p=0.17)
King (2009)	Retrospective cohort	Canada (Calgary, Alberta)	406 patients discharged with a diagnosis of acute myocardial infarction (AMI) and ethnicity determined by surname of Caucasian, Chinese, South Asian, Southeast Asian, or First Nations	Only 34% of Chinese patients, 46% of South Asian patients, and 51% of Southeast Asian patients were fluent in English compared to 99% of Caucasian patients and 92% of First Nations patients (p<0.001) Caucasian patients were more likely to present to the ED by ambulance than other ethnic groups (p<0.001)
Meischke (2012)	Survey	US (King County, Washington)	667 Cambodian adults as identified by surname	Increased measures of acculturation correlated with increased likelihood of calling 911 in an emergency and correlated with increased likelihood of prior CPR training
Ong (2012)	Focus group interviews	US (King County, Washington)	36 adult Chinese speakers with self-identified limited English proficiency recruited from a community center (same sample as Yip 2013)	Chinese LEP adults identified language as an barrier to accessing 911, as well as lack of familiarity with EMS and concerns about delays Knowledge that telephonic interpretation is available was cited as a facilitator to accessing 911 Strategies used to overcome language barriers include finding an English speaker to call 911 and using simple words in English
Sasson (2015)	Focus groups and interviews	US (Denver, Colorado)	64 Latinos in neighborhoods with high rates of cardiac arrest and low rates of bystander CPR	Language barriers were cited as one of six key thematic barriers to calling 911 Participants cited frustration with being placed on hold as a particular barrier and identified bilingual dispatchers as a facilitator
Smith (2010)	Secondary analysis of cohort study	US (Nueces County, Texas)	1,134 Mexican-American and non-Hispanic White adults with ischemic stroke	Spanish-only language was not associated with time to presentation in ED (OR 0.8, CI 0.5–1.3, p=0.4) or arrival via EMS (OR 1.1, CI 0.7–1.7, p=0.7)
Subramaniam (2010)	Survey	US (Detroit, Michigan)	50 limited English proficiency, 50 proficient but non-native English, and 100 native English speaking caregivers in a pediatric emergency department	LEP caregivers were less aware of EMS than native English speaking caregivers (40% unaware of EMS vs. 7% unaware of EMS, p<0.01) LEP caregivers reported less EMS use than native English speaking caregivers (16% had ever called EMS vs. 58% had ever called EMS, p<0.01) 32% of LEP caregivers reported language as a barrier to calling 911
Watts (2011)	Focus group interviews	US (Kansas City, Missouri)	49 Spanish-speaking parents	Familiarity with 911 was high, but parents reported uncertainty about when to access EMS Language was cited as a key barrier to accessing 911, as was fear of repercussions for undocumented immigrants utilizing the services Perceptions of 911 and understanding of EMS logistics was overall good, but Spanish-speaking parents opted to take children directly to an ED
Yip (2013)	Focus group interviews	US (King County, Washington)	36 adult Chinese speakers with self-identified limited English proficiency recruited from a community center (same sample as Ong 2012)	Chinese LEP adults identified reliance on self and community in emergency situations Language barriers and lack of familiarity with 911 were identified as barriers to 911 utilization
Yip (2014)	Survey	US (King County, Washington)	517 Chinese adults as determined by surname and who self-identified as limited English proficiency	When presented with hypothetical scenario of an emergent medical condition for a family member, non-English speaking Chinese adults reported lower likelihood of calling 911 than some-English speaking Chinese adults (p < 0.01)
Publications describing the impact of language barriers on EMS dispatch				
Bradley (2011)	Secondary analysis of randomized controlled trial	US (King County, Washington)	971 cardiac arrest calls	Dispatchers took longer to recognize cardiac arrest with LEP callers compared to non-LEP callers (median 84 seconds vs. 50 seconds, p<0.001) Receipt of bystander CPR was poorer among LEP callers compared to non-LEP callers (OR 0.52, CI 0.29–0.97, p=0.02) Survival to hospital discharge not statistically significantly different (OR 0.49, CI 0.15–1.24, p=0.12)
Heward (2004)	Cross-sectional analysis	UK (London, England)	100 cardiac arrest calls	49% of calls had barriers to performance of dispatcher-assisted CPR and 2% of encounters with barriers was due to language discordance
Lindström (2014)	Qualitative analysis of recorded calls	Sweden (Stockholm County)	100 general 911 calls, 50 of which had agreement on priority level between dispatcher and on-scene providers, 50 of which were determined to be under-triaged by on-scene providers	One-third of calls were by non-native language speakers (22% of calls with agreement on priority level and 10% of calls with under-triage), but language not identified as a barrier to accurate call assessment
Meischke (2010)	Mixed methods	US (King County, Washington)	129 EMS dispatchers; 86 recorded calls with life-threatening complaints and dispatcher-identified LEP callers	70% of dispatchers reported encountering LEP callers almost daily or daily 88% of dispatchers experience these calls as somewhat stressful, stressful, or very stressful 78% of dispatchers believe that language barriers sometimes, often, or always affect medical care While 55% of dispatchers reported that telephonic interpretation is the primary communication strategy that they use with LEP callers, telephonic interpretation was used for only 13% of abstracted calls LEP callers less likely than non-LEP callers to have BLS and ALS resources simultaneously dispatched despite similar chief complaints (20% vs. 38%, p=0.01)
Meischke (2013)	Case-control study	US (King County, Washington)	272 EMS calls with a language barrier as identified by dispatcher matched to 272 calls without a language barrier during a 4-month period	Increased time to dispatch of BLS resources (p<0.001) and ALS resources (p=0.008) with language barrier Increased likelihood of on-scene change in resources for calls with language barrier (OR 2.36, CI 1.29–4.33, p=0.006) Connecting to a telephonic interpreter required a mean of 158 seconds
Meischke (2014)	Randomized-controlled trial	US (King County, Washington)	139 self-identified limited-English proficient adults with primary languages of Mandarin, Cantonese, or Spanish	In a cardiac arrest simulation in which participants called a simulated 911 dispatcher for assistance, including bystander CPR instructions, use of a telephonic interpreter increased time to first compressions by nearly 2 minutes compared to a standardized language protocol or a protocol in which the telecommunicators could rephrase the protocol language (mean 288 s vs. means 176 s and 168 s, p<0.001) Participants reported better understanding of dispatcher instructions with interpreter use, but there was no improvement in quality of CPR with interpreter use There was no difference in time to first compressions or quality of CPR for the protocol in which telecommunicators could rephrase the protocol language as compared to a standardized language protocol
Publications describing the impact of language barriers on EMS care in the field				
Cottrell (2014)	Focus group interviews	US (Multnomah County, Oregon)	40 paid and volunteer EMS providers	Language barriers cited as one child and family-level factor contributing to prehospital pediatric safety events
Grow (2008)	Cross-sectional analysis	US (Minnesota)	15,620 reports that identified a prehospital delay in the Minnesota State Ambulance Reporting system database during 18-month period	Language barriers were the second most commonly cited cause of prehospital delay (13% of delays) Time on-scene for encounters identified as delay due to language barrier was actually shorter than the no delay encounters (mean time on scene of 16.00 minutes vs. 21.98 minutes)
Shaw (2014)	Focus groups	UK (East Midlands region, England)	17 paramedics	Patient language identified as one type of communication barrier to adherence to prehospital asthma guidelines
Sterling (2013)	Cross-sectional analysis	US (New Jersey)	11,249 EMS encounters for chest pain excluding cardiac arrest	Language discordance associated with shorter on-scene times for chest pain encounters (mean 8.93 minutes for language discordant vs. 9.78 for language congruent, p<0.0001)
Weiss (2014)	Retrospective double cohort study	US (Albuquerque, New Mexico)	59 limited-English proficiency patients and 100 English-proficient patients as determined by ability to sign an English-only form	Language barriers not associated with differences in on-scene times, transport times, number of interventions, medications, or pain scores when corrected for age and gender

*EMS,* emergency medical services; *CPR,* cardiopulmonary resuscitation; *LEP,* limited-english proficiency

*ED,* emergency department; *OR,* odds ratio; *CI,* confidence interval; *LEP,* limited-english proficiency; *EMS,* emergency medical services

*EMS,* emergency medical services; *LEP,* limited-english proficiency; *BLS,* basic life support; *ALS,* advanced life support; *OR,* odds ratio; *CI,* confidence interval; *CPR,* cardiopulmonary resuscitation

*EMS,* emergency medical services
